# The Effect and Relative Importance of Neutral Genetic Diversity for Predicting Parasitism Varies across Parasite Taxa

**DOI:** 10.1371/journal.pone.0045404

**Published:** 2012-09-26

**Authors:** María José Ruiz-López, Ryan J. Monello, Matthew E. Gompper, Lori S. Eggert

**Affiliations:** 1 Department of Fisheries and Wildlife Sciences, University of Missouri, Columbia, Missouri, United States of America; 2 Division of Biological Sciences, University of Missouri, Columbia, Missouri, United States of America; University of Utah, United States of America

## Abstract

Understanding factors that determine heterogeneity in levels of parasitism across individuals is a major challenge in disease ecology. It is known that genetic makeup plays an important role in infection likelihood, but the mechanism remains unclear as does its relative importance when compared to other factors. We analyzed relationships between genetic diversity and macroparasites in outbred, free-ranging populations of raccoons (*Procyon lotor*). We measured heterozygosity at 14 microsatellite loci and modeled the effects of both multi-locus and single-locus heterozygosity on parasitism using an information theoretic approach and including non-genetic factors that are known to influence the likelihood of parasitism. The association of genetic diversity and parasitism, as well as the relative importance of genetic diversity, differed by parasitic group. Endoparasite species richness was better predicted by a model that included genetic diversity, with the more heterozygous hosts harboring fewer endoparasite species. Genetic diversity was also important in predicting abundance of replete ticks (*Dermacentor variabilis*). This association fit a curvilinear trend, with hosts that had either high or low levels of heterozygosity harboring fewer parasites than those with intermediate levels. In contrast, genetic diversity was not important in predicting abundance of non-replete ticks and lice (*Trichodectes octomaculatus*). No strong single-locus effects were observed for either endoparasites or replete ticks. Our results suggest that in outbred populations multi-locus diversity might be important for coping with parasitism. The differences in the relationships between heterozygosity and parasitism for the different parasites suggest that the role of genetic diversity varies with parasite-mediated selective pressures.

## Introduction

While most individuals in a host population carry few or no macroparasites of a particular species, a few individuals harbor high numbers of parasites [1]. The causal factors underpinning this general pattern, which typically fits a negative binomial distribution, differ as a function of the parasitic species under study and the environmental context of the host-parasite interaction. Nonetheless, it is generally recognized that this variation is due to the complex interaction of factors extrinsic and intrinsic to the host, including where a host lives, temporal variability in host-parasite interactions, and variability in host susceptibility, which depends on factors such as age, sex, body condition, or genotype.

A primary focus of many theoretical and empirical studies has been to understand the linkages between the genetic diversity of host populations and their susceptibility to pathogens [2]–[8]. The association between reduced levels of genetic diversity and increased prevalence or abundance of pathogens, termed “the monoculture effect” [9]–[10], has been demonstrated in studies of both agricultural and wild species [11]–[13]. In fact, pathogen-mediated selection has been proposed as a major underlying mechanism that promotes the accumulation and maintenance of genetic diversity in host populations through selection against common genotypes (Red Queen Hypothesis, [3]) or homozygous genotypes [14]–[16]. For example, Bérénos et al. [17] showed that lines of Red Flour Beetle (*Tribolium castaneum*) that had coevolved with a microsporidian parasite had higher levels of heterozygosity than control lines that had not coevolved with the parasite. Underlying cross-population and interspecific relationships between genetic variability and parasitism is the differential susceptibility of individuals. Within populations, variance in host genetic diversity (measured as heterozygosity) can be an important predictor of parasitism. In wild populations it has been shown that individuals with lower heterozygosity may have higher infection frequencies and greater morbidity [18]–[21]. This extreme has been especially explored in the contex of inbreeding. When inbreeding occurs, the increase in the frequency of alleles that are identical by descent generates correlations in the extent of heterozygosity or homozygosity across loci throughout the genome (i.e. identity disequilibrium) [22]–[24]. Therefore, inbred individuals have a higher probability of homozygosity at all loci, including genes involved in disease resistance [20], and parasites would select against these hosts, ultimately favouring increased genome-wide genetic diversity in the host population [12], [25].

Associations between individual host genetic diversity and parasitism have frequently been studied using multi-locus heterozygosity at neutral genetic markers (microsatellites) as a proxy for genome-wide diversity [i.e heterozygosity-fitness correlations (HFC)]. Several studies have shown that the parasite HFCs were a consequence of inbreeding depression [18]–[19]. However, it is not uncommon to find significant HFCs in non-inbred populations [14], [21], [26], although in those cases multi-locus heterozygosity is thought to be a poor predictor of genome-wide diversity [27]–[29]. The alternative explanation for these findings is that instead of inbreeding that affects the whole genome, linkage disequilibrium (i.e. non-random associations of different loci in the gamete) may occur between neutral and functional loci [23], [27], [30] due to the high polymorphism of the microsatellites that favor finding linkages between alleles and candidate genes ([31]; but see [24] and [32]). Although the exact underlying mechanisms at functional loci that cause these HFCs are still under debate, overdominance (i.e. heterozygote advantage; [23], [30], [33]) is frequently proposed as a likely explanation, especially for parasite resistance loci [14], [15].

However, it has been difficult to generalize about correlations between neutral genetic variability and measures of parasitism since such patterns are sometimes equivocal. Results vary with the species of parasite studied and its fitness effects on the host as well as the general level of inbreeding of the host population. There are several examples where less heterozygous individuals were more susceptible to parasite infection [18], [19], [34]. There are also instances in which no association between genetic diversity and parasitism was found [4], [8], [35] or where such correlations were limited to loci that are physically close to immune response candidate genes [36]. Yet, as with other HFCs, the role of genetic diversity in predicting parasite loads is environment and context dependent [26]. Parasitism is influenced by many abiotic and non-genetic biotic factors that determine exposure and susceptibility. For example, if the fitness consequences of a parasite are mild, the role of genetic diversity relative to these other factors may be weak and difficult to detect. Indeed, in some cases genetic diversity has been found to play a role only for young individuals, for whom the effects of the studied parasite on fitness were stronger [37]. In addition, as with inbreeding depression, it is possible that the role of genetic diversity is stronger under stressful environmental conditions [18], [25]. Finally, despite the fact that not all parasites influence hosts equally, and that HFCs may differ as a function of the examined parasitic taxon [35], [38], most studies are carried out for a single parasite without consideration of the potential for differences in the effect of genetic diversity on susceptibility to different parasite species.

Here we address these issues by considering the relative importance of neutral genetic variability when compared to non-genetic factors that have been shown to be important in predicting parasitism. We focused on outbred, free-ranging populations of raccoons (*Procyon lotor*) for which we have collected data on behavioral, population, and habitat ecology and for which observational and experimental studies have identified non-genetic predictors of parasitism [39]–[43]. For these populations, non-genetic predictors of parasitism include both factors extrinsic (annual and seasonal variability, location of the host population and contact rates) and intrinsic to the host (age, sex and body condition). We recently estimated the genetic diversity by measuring individual genetic diversity of individuals in these populations and observed that even small differences in the neutral genetic variability of individuals comprising these populations are associated with the animal’s ability to overcome infection by canine distemper virus (CDV), a pathogen that causes high rates of mortality in raccoons [44]. Thus, genetic variability in combination with these non-genetic factors may enhance our ability to predict the extent of parasitism.

In this study we analyze data on infection by macroparasites, including two species of ectoparasites (ticks and lice) and a collective measure of the extent of infection by internal macroparasites (endoparasite species richness). While ecto- and endoparasites may have important effects on host fitness, presumably these effects are less extreme and more variable than that of CDV, which can directly kill hosts. Using an information theoretic approach we selected the best models for each parasite and assessed whether individual genetic diversity is a significant predictor of the extent of ecto- and endoparasitism when placed in the context of non-genetic factors already identified as important predictors [39]–[42] and determined whether the relationship between genetic diversity and parasitism differs among parasitic species or groups. We expected to find an overall negative relationship between levels of genetic diversity and parasite load, i.e, more heterozygous individuals will present fewer parasites. We also expected the relationship between genetic diversity and parasite load to differ across parasite taxa, being stronger for those parasites that may have greater fitness consequences and are known to trigger immune responses because there are higher chances of these pathogens to be imposing selective pressures on the host. Endoparasites, for example, are known to trigger an immune response [45], thus they may have larger effects on host fitness than ectoparasites and we predicted that they would have a stronger relationship with genetic variability. Yet some ectoparasites also interact with hosts more closely than others, and thus we predict that female ticks that have remained on a host long enough to draw a blood meal and become replete will show a stronger relationship with genetic variability than ticks that have only recently attached to hosts or chewing lice that feed on skin debris and may not trigger an immune response [46]–[47]. We also tested whether such relationships are best explained by single locus effects or genome-wide neutral genetic diversity. Since HFCs in outbred populations have been frequently associated with strong effects of single loci [30], [48], we predicted that the relationship is due to single locus effects.

## Materials and Methods

### Ethical Statement

Research was carried out under Missouri Department of Conservation permit #12869, which specifically approved this study, and University of Missouri Animal Care and Use Protocol #3927.

### Sampling Hosts

**Figure 1:**
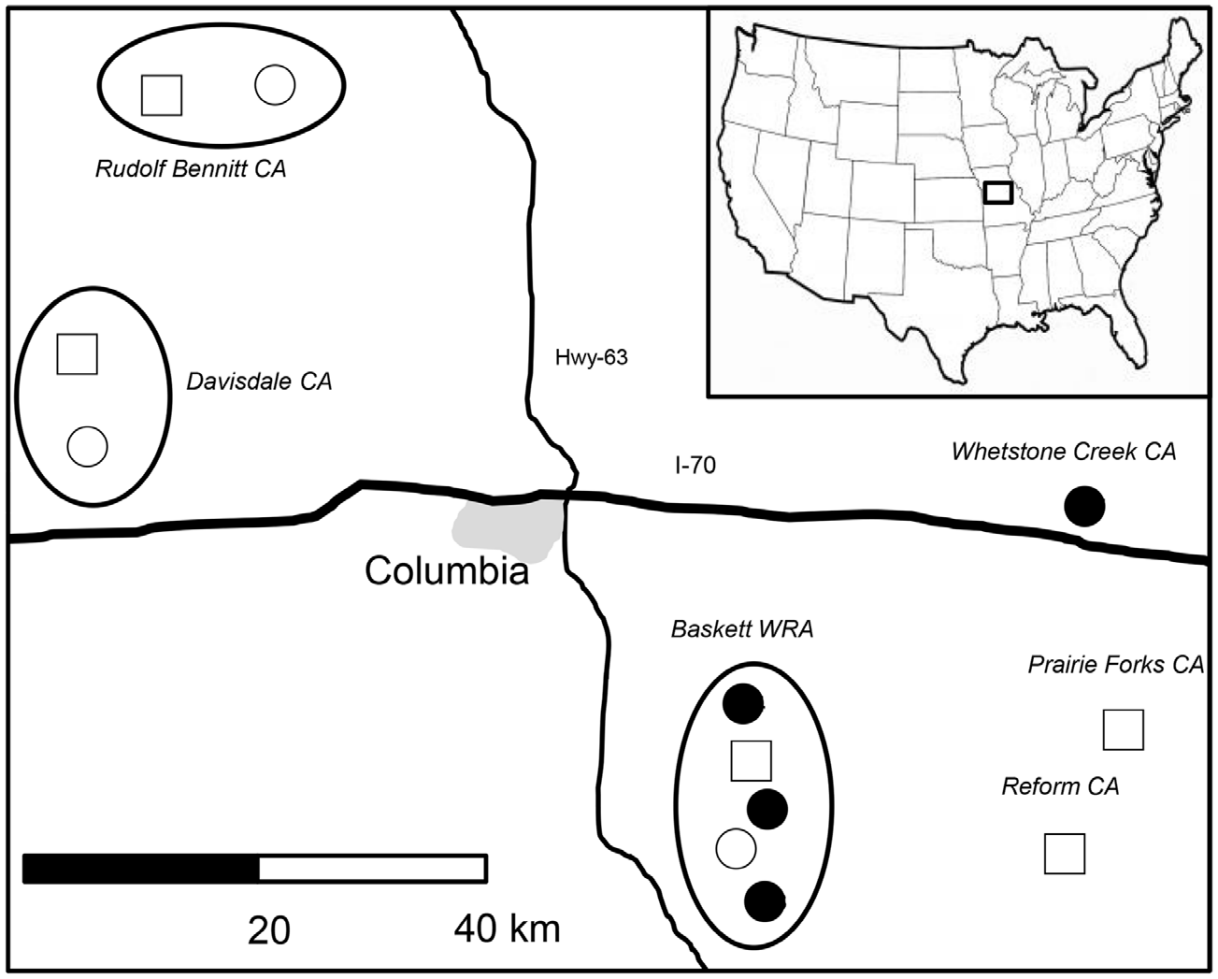
Location of the 12 study sites contained within the 6 source areas in central Missouri, USA. Source areas were defined based on Fst assessments as Baskett, Rudolf Bennitt, Davisdale, Reform, Prairie Forks and Whetstone Creek. Location and treatments of each site are indicated by closed circles (control sites which did not receive supplemental food and thus raccoons did not aggregate), open circles (sites which received food, but food was dispersed so as to not to cause raccoons to aggregate) or open squares (sites which received supplemental food at a single site so as to cause raccoons to aggregate).

Raccoons were sampled between 2006 and 2007 at 12 forested sites located within 60 km of Columbia, Missouri, USA (Figure 1). Details of the sites, raccoon populations and associated macroparasite communities, and host and parasite sampling protocols are given elsewhere [39]–[43]. In brief, all sites had similar raccoon population densities, and measures of genetic variability indicate that these populations are highly variable and outbred [41]–[44]. Sites received different experimental treatments as part of a study that measured the effects of differential contact rates and food provisioning on parasite communities [41], [42]. One of the following three experimental treatments was randomly assigned to sites within geographically defined blocks: 1) a permanent feeding station stocked with 35 kg/wk of dried dog food at a single location to aggregate raccoons (n = 5 sites); 2) the same quantity of food, placed at highly dispersed and temporally variable locations to control for the effects of food addition without aggregating hosts (n = 3 sites), or 3) no food additions (n = 4 sites). Prior work on these host populations found that aggregation increased tick abundance and decreased lice abundance [41], while supplemental food decreased the number of indirectly transmitted endoparasites [42].

Raccoons were trapped for ≥10 days at each site two to three times per year between March and November. Individuals were anaesthetized and ear-tagged, weighed, sexed, measured, and aged [41], [49] as kits (0–5 months) or age class I (6–14 months), II (15–38 months), III (39–57 months) or IV (>58 months). Data from kits, which formed only a small portion of the sampled individuals, were excluded from subsequent analyses because they were generally free of ecto- and endoparasites [39], [40], [42]. Residuals from a linear regression of body mass on body size were used to assess the relative body condition of each individual [50]. Hair samples and blood samples, collected via femoral venipuncture and placed in EDTA, were stored at −20°C. Animals were released at the site of capture following recovery from anesthesia.

### Sampling Parasites

We focused on two species of ectoparasites: the American dog tick *Dermacentor variabilis*, and the chewing louse *Trichodectes octomaculatus* [39]–[41]. *Dermacentor variabilis* is a 3-host metastriate tick that occurs on raccoons while feeding or mating. Adult *D. variabilis* are large (3–5 mm in length) and readily found and identified without magnification. We focused on adult ticks present between April and August, the primary period when this species parasitizes raccoons at our study area [39], and quantified abundance by a thorough search of the entire body. Ticks were classified as non-replete (i.e. males and females that are not engorged with blood), semi-replete (females that have entered the slow feeding stage but are not yet fully engorged), or replete (female is fully engorged, indicating that mating has occurred and the animal has entered a rapid feeding phase). Only ticks in the non-replete and replete groups were included in the analyses since they constitute two well differentiated categories. Lice were sampled via 10 strokes with a flea comb from the base of the neck to the base of the tail on the dorsal region. Lice were placed in sealed plastic bags and frozen until transfer to the laboratory where they were identified to species and counted under a dissecting scope. To avoid handling effects on ectoparasite abundance and to facilitate a similar likelihood for finding ectoparasites for each host, only the first capture events for each host were included in analyses.

For sampling endoparasites, fresh feces were collected from within or below traps, homogenized, and stored in 10% formalin. The presence of endoparasite species was based on identification of ova and oocysts isolated by fecal flotation procedures using sugar and zinc sulfate centrifugation techniques. Additional methodological details and citations for endoparasite species descriptions and identification are provided elsewhere [42]. Based on the presence/absence data, we calculated prevalence of each endoparasite species and endoparasite richness for each host, which is a proxy of host endoparasite burden. Only one randomly chosen fecal sample per individual host was included in the analyses. Although using additional samples may give a more accurate parasite species richness index for an animal, this would have resulted in a disproportionately larger sampling effort for recaptured raccoons.

### DNA Extraction and Genotyping

Total genomic DNA was extracted from blood samples using DNeasy Blood and Tissue Kits (Qiagen, Valencia, CA, USA) with the manufacturer’s protocol and from hair samples using InstaGene Matrix kits (BioRad, Hercules, CA, USA) following [51]. Each individual was genotyped at 15 unlinked nuclear microsatellite loci developed for raccoons: PLM01, PLM03, PLM05, PLM06, PLM07, PLM08, PLM09, PLM10, PLM11, PLM12, PLM13, PLM14, PLM15, PLM16 and PLM17 [52]. A total of 203 individuals were previously genotyped at 12 of these loci [44]; these individuals were genotyped for the 3 additional loci (PLM1, PLM3 and PLM17). An additional 177 individuals were genotyped using a multiplex approach, as were 24 of the initial 203 individuals to ascertain genotyping consistency between the two datasets. The 15 microsatellites were co-amplified in three multiplex PCRs following the Multiplex PCR Kit (Qiagen) protocol for 40 cycles (blood extracts) or 45 cycles (hair extracts) and a 60°C annealing temperature. Reactions for DNA extracted from blood were prepared in a final volume of 10 µl containing 1 µl DNA (15–20 ng), 1X Multiplex Master Mix, 0.065 µM each primer, and 0.8 mg/ml BSA. Reactions for the DNA extracted from hair were prepared in a final volume of 12.5 µl containing 3 µl DNA, 1X Multiplex Master Mix, 0.1 µM each primer, and 0.8 mg/ml BSA. Fragment length analyses were performed on an ABI 3730 DNA Analyzer (Applied Biosystems, Foster City, CA, USA), and alleles were scored using GENEMARKER 1.5 (SoftGenetics, State College, PA, USA). We repeated analyses of 35% of blood samples to calculate genotyping error rate. Because DNA derived from hair samples may have low DNA quality and quantity, heterozygous genotypes were confirmed in at least two separate reactions and homozygous genotypes were confirmed in at least three separate reactions.

### Genetic Analyses

We tested for deviations from expected genotype frequencies under Hardy-Weinberg equilibrium and for linkage disequilibrium between all pairs of loci using GenePop 3.4 [53]. The mean number of alleles and mean expected and observed heterozygosity values were calculated with the program Arlequin 3.1 [54]. The probability of null alleles was estimated using Microchecker 2.2.3 [55]. Using the Excel-macro IRmacroN4 (www.zoo.cam.ac.uk/zoostaff/amos) we calculated three measures of individual genetic diversity: standard multilocus heterozygosity (sMLH; [18], internal relatedness (IR; [56]) and heterozygosity weighted by locus (HL; [57]). sMLH represents general levels of heterozygosity and controls for the number of loci genotyped [18]. IR and HL are measures of homozygosity but differ in how they are calculated. IR weights homozygotes for rare alleles more heavily than homozygotes for common alleles since the former are more likely derived from related parents, and thus gives a measure of the extent of inbreeding [56]. HL weights contribution of each locus to overall homozygosity in proportion to their allelic variability [57]. To calculate these measures we determined if there was a strong variance across years or sites since these measures have to be calculated at the population level. An Analysis of Molecular Variance (AMOVA) showed no significant partition of variance across years and population structure analyses showed that in spite of isolation by distance differences among some of the sites, these comprise a single population (overall F_ST_ = 0.008). Thus, sMLH, IR and HL were calculated based on gene frequencies pooled across all years and sites. These measures are frequently highly correlated and testing all of them could lead to pseudoreplication [26]. Therefore we calculated pairwise Pearson correlation coefficients to assess the relationship of the three metrics. As expected, the three measures of genetic diversity were highly correlated (r_SMLH-IR_ = −0.980; r_IR-HL_ = 0.980; r_HL-SMLH_ = −0.992; all p<0.001). Therefore, since HL and sMLH had a correlation coefficient higher than 0.99, we excluded sMLH from further analyses. Simulations suggest that HL may be better correlated with inbreeding coefficient and genome-wide homozygosity than IR in populations that present levels of heterozygosity greater than 0.6. However, this only occurred in study populations with high genetic structure and admixture, and the differences were clearer when ≥50 markers were used [57]. Because the population under study does not fulfil all these assumptions, we carried out analyses using both IR and HL. Both IR and HL were normally distributed, and thus we used one-way analyses of variance (ANOVA) to test for differences in individual genetic diversity across populations and years, and among age and sex classes.

### Effects of Multi-locus and Single-locus Heterozygosity on Parasitism

For assessments of genome-wide genetic diversity effects, we used information-theoretic model selection [58] to identify the importance of an individual’s genetic diversity relative to other factors known to predict the likelihood or extent of parasitism. To assess whether individual genetic diversity is a significant predictor of parasitism when placed in the context of other factors already identified as important predictors of infection, we conducted a two-stage modeling approach. We first identified the best non-genetic model that included predictors extrinsic to the host (site, month, year, aggregation, food supplementation) and predictors intrinsic to the host (age, sex, body condition), and then evaluated whether the model was improved through the addition of individual genetic diversity measures. In both stages we calculated Akaike’s information criterion corrected for small sample size (AIC_c_) and then calculated the differences between the best approximating model (the model with the lowest AIC_c_) and all the other models (ΔAIC_c_), as well as Akaikés weights (*W*_i_) and the evidence ratio (Δ_i_), for each model in the candidate set [58].

In the first stage, selection of the non-genetic terms for inclusion in the models was carried out *a priori* based on previous studies that identified important predictors of parasitism for each parasitic group [39]–[42]. In the second stage we selected all the models from the first stage that were within 2 ΔAIC_c_ units of the best-fitting model and compared them to new models that included genetic diversity (both the variable and its quadratic term to control for potential non-linear relationships; [59]) as potential explanatory variables. Since significant population structure can lead to spurious significant HFCs [60], we controlled for the potential effects of the isolation-by-distance among the 12 study sites by including the 6 source areas (as defined by F_ST_ assessments; Ruiz-Lopez et al. unpublished data) as a covariate in all models that collectively included the 12 sites (Figure 1).

To compare the effect of genetic diversity and its quadratic term we standardized these continuous predictors by centering and dividing by 2 standard deviations (SDs) to force a mean of 0 and SD of 0.5. We calculated model averaged estimates and odds ratios (±95% C.I.) for each variable in the 90% confidence set of models (i.e. inclusion of all parameters in top models that collectively sum to w = 0.90). All analyses were conducted using R.v.13.1 (R Development Core Team 2011); the libraries MASS, and AICcmodavg were used to carry out model selection and the library arm to standardize the variables. Ectoparasite abundance was analyzed using generalized linear models with a negative binomial distribution and a log link function [41]. For *D. variabilis,* we analyzed non-replete and replete ticks separately because the importance of factors intrinsic and extrinsic to the host differs for these tick classes [39]. Endoparasite species richness was analyzed using a general linear model with a normal distribution [42].

For cases where the best models included a measure of genetic diversity we assessed the potential effects of single-locus heterozygosity. To do so, we selected the top model and replaced the multilocus heterozygosity measure with the single locus heterozygosity at each marker incorporated as a binary variable (0 if homozygous and 1 if heterozygous). We compared both models (the one fitting the multilocus heterozygosity and the one fitting the single locus effects) using an F-test to determine which of the two models explained significantly more variance [24]. Only individuals with complete genotypes were included in these analyses. From the models we calculated the effect size as the partial correlation coefficient [61]. We used a binomial sign test to investigate whether positive and negative effects occurred equally. We also tested whether metrics of genetic diversity (number of alleles, and expected and observed heterozygosity) were associated with the single locus effect size by regressing the effect size on genetic diversity.

## Results

### Parasite Prevalence and Abundance

Across all years tick prevalence was 96%, and individual abundance ranged from 0 to 142 with a mean of 23.43 ticks per individual (n = 259; Table S1). Prevalence and abundance differed for the two tick categories, with non-replete ticks presenting higher prevalence and mean abundance (Prevalence_non-replete_ = 0.95; Prevalence_replete_ = 0.55; Abundance_non-replete_ = 21.43; Abundance_replete_ = 1.99). Louse prevalence was 0.52 and mean louse abundance ranged from 0 to 55 with a mean of 3.03 (n = 307). The endoparasite community of raccoons at the study sites comprised 16 taxa [42]. Prevalence of each endoparasite species ranged from 0.14 to 0.89 (Table S1).

### Descriptive Genetic Analyses

Of 380 raccoons sampled between 2006 and 2007, 94.7% were genotyped at ≥12 markers, and were included in subsequent analyses. Mean observed heterozygosity was 0.783 (SD = 0.094), mean expected heterozygosity was 0.793 (SD = 0.090), and mean number of alleles per locus was 11.3 (SD = 3.2; range = 6–19; Table S2). Loci PLM7 and PLM12 were marginally significant (p-value <0.03) for a heterozygosity deficit after Bonferroni correction. Whereas PLM7 did not present evidence of null alleles, the probability of null alleles at locus PLM12 was close to 0.05 (probability of null alleles Oosterhout = 0.047). Therefore, we carried out all HFC analyses with and without the PLM12 locus, but kept PLM7 in all the analyses. While inclusion of PLM12 locus did not significantly affect the results, to be conservative we excluded data for this locus, and therefore subsequent results are based on a final panel of 14 microsatellite loci. Over all possible pairs, no pair of loci showed significant linkage disequilibrium after Bonferroni correction. Mean sMLH was 1.035 (SD = 0.141), mean IR was 0.009 (SD = 0.130), and mean HL was 0.202 (SD = 0.108). Neither IR nor HL showed significant differences across experimental treatments, sites, years, age or sex categories.

IR and HL yielded similar results when included in the models, probably due to the lack of strong genetic structure among our populations and because we used 14 markers. Therefore, below we present only the IR-based results.

### Effect of Multilocus Heterozygosity on Ectoparasite Abundance

**Figure 2:**
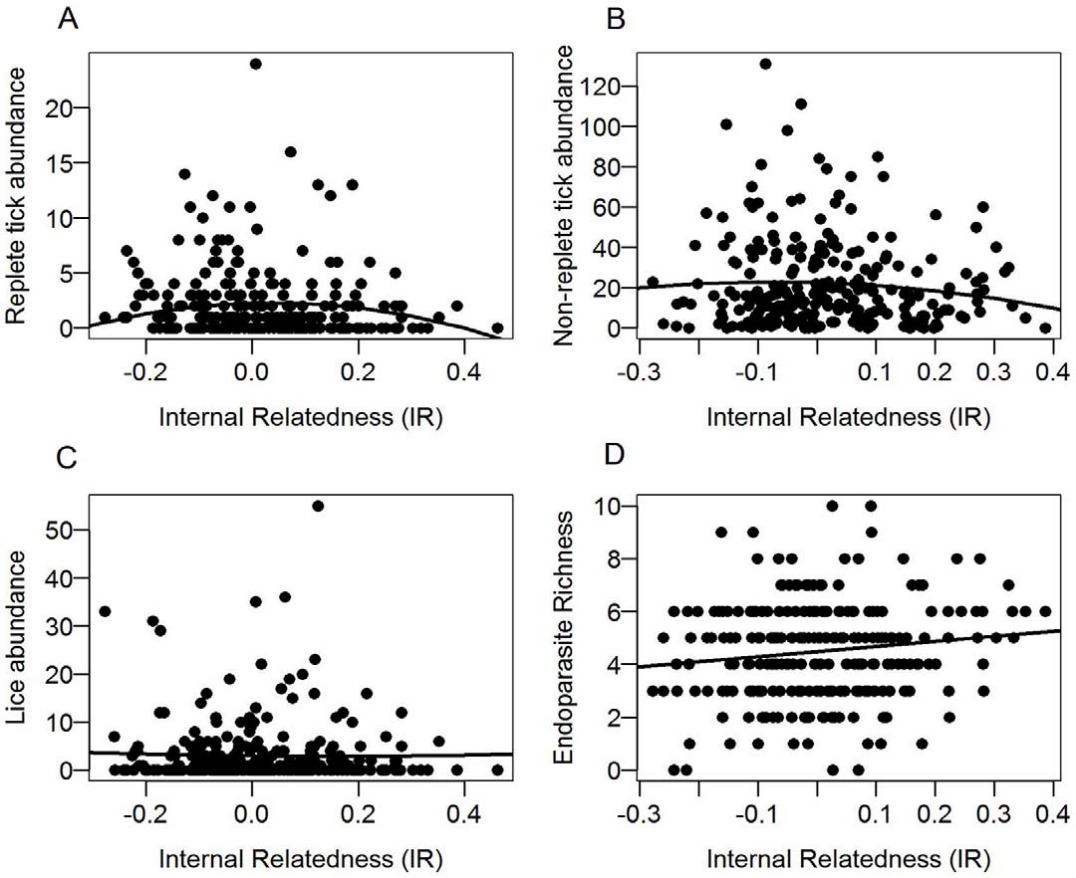
Relationship between multi-locus genetic diversity (internal relatedness, IR) and individual parasite load. A- replete tick abundance; B- non-replete tick abundance; C- lice abundance and D- endoparasite richness.

The results of adding genetic diversity to the best non-genetic models differed for lice and non-replete and replete ticks (Table 1). Genetic measures were important in explaining tick abundance, especially for replete ticks, but not for explaining lice abundance. For replete ticks, the best-fitting non-genetic model included the parameters aggregation, food, and month. This model garnered the majority of model support (*Wi* = 0.63) and no other model fell within 3 ΔAIC units (Table S3a). In Stage 2 of the replete tick analyses, however, the top non-genetic model garnered less support (*Wi* = 0.18) and the top models contained genetic terms (*Wi* = 0.73) (Table 1a). Interestingly, the top two models included the quadratic term (IR^2^) both alone or together with the linear IR term, revealing an underlying curvilinear association between parasite loads and IR. Model average estimates and odd ratios showed that this relation was negative (β = −0.52, odds = 0.59), indicating that moderately heterozygous individuals had, on average, more parasites (Figure 2a). Model averaged estimates for IR^2^ (-0.52) and aggregation (0.60) were similar in magnitude and in both cases the 95% CI did not overlap 0, collectively indicating that their effects on tick abundance were significant and of similar relative importance (Table 2). Model estimates for IR were smaller and the 95% CI, although highly skewed towards positive values, overlapped 0 (Table 2). The three factors that had a higher effect on replete tick abundance were area (with higher abundance in Davisdale CA, Rudolf Bennitt CA and Whetstone Creek CA), month (with higher abundance in July), and aggregation (with higher abundance in aggregated sites) (Table 2).

**Table 1:** Ranking of models estimating abundance of (a) replete and (b) non-replete ticks (n = 259) and (c) lice (n = 307) in raccoons, including non-genetic and genetic terms.

Model	k	ΔAICc	W*_i_*	Δ*i*	log (l)
(a) ***Replete ticks***					
Aggregation+Food+Month+IR+IR^2^+Area	15	0.00	0.43	0.00	−458.53
Aggregation+Food+Month+IR^2+^Area	14	0.74	0.30	1.45	−460.03
Aggregation+Food+Month+Area	13	1.69	0.18	2.33	−461.62
(b) ***Non-replete ticks***					
Month+Year+Aggregation+Food+Age+Sex+ Area	18	0.00	0.34	0.00	−984.55
Month+Year+Aggregation+Food+Area	14	0.74	0.23	1.45	−989.48
Month+Year+Aggregation+Food+Age+Sex+IR^2^+Area	19	2.22	0.11	3.04	−984.50
Month+Year+Aggregation+Food+Age+Sex+IR+Area	19	2.22	0.11	3.04	−984.50
Month+Year+Aggregation+Food+IR^2^+Area	15	2.92	0.08	4.31	−989.45
Month+Year+Aggregation+Food+IR+Area	15	2.99	0.08	4.45	−989.48
(c) ***Lice***					
Aggregation+ Age+Sex+Age*Sex	10	0.00	0.53	0.00	−579.82
Aggreg+Food+Age+Sex+Body Condition+Age*Sex	12	1.80	0.21	2.46	−578.57
Aggregation+ Sex	4	2.23	0.17	3.04	−587.24

Analyses were conducted separately for each species and category within species. Models included represent the 90% confidence set (∑weight >0.90) used to calculate the model averages. k = number of model parameters, *W*_i = _Akaikés weight, Δ_i_ = evidence ratio, log (l) = log-likelihood value.

**Table 2:** Model averaged estimates and odds ratios of parameters included in the 90% confidence set of models (∑weight >0.90) used to estimate abundance of non-replete and replete *D. variabilis*.

	Replete *D. variabilis*			Non-replete *D. variabilis*		
Predictor	β(±95%CI)	SE	Odds(±95%CI)	β (±95%CI)	SE	Odds(±95%CI)
IR	0.40(−0.05,0.85)	0.23	1.49(0.95,2.34)	−0.02(−0.23,0.18)	0.11	0.98(0.79,1.20)
IR^2^	−0.52(−1.01, −0.03)	0.25	0.59(0.36,0.97)	−0.03(−0.25,0.18)	0.11	0.97(0.78,1.20)
Area (Davis)	0.82(0.15,1.49)	0.34	2.27(1.16,4.43)	1.53(1.17, 1.89)	0.19	4.61(3.22,6.61)
Area (PF)	0.15(−0.79,1.09)	0.48	1.18(0.46,3.03)	1.31(0.80,1.81)	0.26	3.71(2.22,6.11)
Area (R)	1.10(0.32,1.87)	0.39	3.00(1.37,6.48)	1.32(0.86,1.77)	0.23	3.74(2.36,5.87)
Area (Ref)	0.17(−0.79,1.14)	0.49	1.18(0.45,3.13)	0.83(0.30, 1.36)	0.27	2.29(1.35,3.90)
Area (Whet)	0.89(0.16,1.62)	0.37	2.43(1.17,5.05)	0.39(−0.03,0.81)	0.21	1.47(0.97,2.25)
Month (August)	−0.18(−1.02,0.67)	0.43	0.83(0.36,1.95)	−0.87(−1.34, −0.41)	0.24	0.42(0.26,0.66)
Month (July)	0.85(0.17,1.54)	0.35	2.34(1.18,4.66)	0.95(0.57,1.33)	0.19	2.59(1.77,3.78)
Month (June)	0.57(−0.22,1.37)	0.41	1.77(0.80,3.93)	0.71(0.28,1.14)	0.22	2.03(1.32,3.13)
Month (May)	0.58(−0.26,1.42)	0.43	1.78(0.77,4.14)	0.45(−0.02,0.91)	0.24	1.57(0.98,2.48)
Aggregation (Yes)	0.60(0.09,1.10)	0.26	1.82(1.09,3.00)	0.36(0.08,0.64)	0.14	1.43(1.08,1.89)
Food (Yes)	−0.14(−0.88,0.60)	0.37	0.87(0.41,1.82)	−0.41(−0.81, −0.01)	0.20	0.66(0.44,0.99)
Year (2007)	–	–		−0.12(−0.35,0.11)	0.12	0.88(0.70,1.12)
Sex (Male)	–	–		0.31(0.10,0.52)	0.11	1.36(1.11,1.68)
Age II	–	–		−0.07(−0.34,0.21)	0.14	0.93(0.71,1.23)
Age III	–	–		0.12(−0.19,0.43)	0.16	1.13(0.83,1.54)
Age IV	–	–		0.05(−0.27,0.37)	0.16	1.05(0.76,1.45)

The parameters Area (Baskett), Year (2006), Food (No) and Sex (Female) were used as the reference level and model average set to 0.

Davis = Davisdale CA, PF = Prairie Forks CA, R = Rudolf Bennitt CA, Ref = Reform CA, Whet = Whetstone Creek CA.

The top non-genetic models for non-replete ticks included temporal terms (month, year), treatment category (aggregation, food), and factors intrinsic to the host (age, sex) (Table S3b). This model (now including area to account for the fine genetic differences across the different conservation areas) remained the top model when genetic variability was included as a potential explanatory metric. The top model that included genetic diversity was the third ranked model (ΔAICc = 2.2) (Table 1b). Estimates of β for IR and IR^2^ were close to and overlapped 0. Model average estimates indicated that the most important factors were area and month followed by food, aggregation and sex. By comparison, year, age and genetic diversity had less relative importance (Table 2, Figure 2b).

For lice, the top non-genetic model, which included host aggregation, age, sex, and an age*sex interaction (Table S3c), was not improved when measures of genetic diversity were included as additional potential explanatory parameters. In fact, no models that included IR or IR^2^ fell within the 90% confidence set of models of the top predictive model (Table 1c). The top model that included a measure of genetic diversity differed by 8.04 AICc units from the best fitting model, and given the low weight of evidence in support of this model (*Wi* = 0.01), no genetic parameter was in the final model averaged results (Table S4).

### Effect of Multilocus Heterozygosity on Endoparasite Richness

The top non-genetic model predicting endoparasite species richness comprised age and year (*Wi* = 0.35). Two additional models had moderate support (*Wi* = 0.21) and included the parameters age, sex, year, and food, all of which were used in the second modeling stage which incorporated genetic parameters and the six source areas (Table S3d). Adding IR to the best non-genetic models predicting endoparasite richness improved the fit of these models (Table 3). The top two models, with a combined *Wi* = 0.40, included IR and most of the models within the 90% confidence set of models included either IR or the quadratic term. The top model that comprised solely non-genetic terms differed by ΔAICc = 0.91, and the top model identified during the first stage of the analyses (age + year) differed by ΔAICc = 8.44 from the best model that included genetic variability.

**Table 3:** Ranking of models estimating endoparasite richness in a raccoon population (n = 250) including non-genetic and genetic terms.

Model	k	ΔAICc	W*_i_*	Δ*_i_*	log (l)
Age+Food+Year+Sex+IR+Area	14	0.00	0.22	0.00	−489.61
Age+Food+Year+IR+Area	13	0.46	0.18	1.26	−490.96
Age+Food+Year+Sex+Area	13	0.91	0.14	1.58	−491.19
Age+Food+Year+Area	12	1.28	0.12	1.89	−492.48
Age+Food+Year+Sex+IR+IR^2+^Area	15	2.12	0.08	2.89	−489.54
Age+Food+Year+Sex+IR^2+^Area	14	2.27	0.07	3.12	−490.75
Age+Food+Year+IR+IR^2^+Area	14	2.59	0.06	3.65	−490.91
Age+Food+Year+IR^2^+Area	13	2.71	0.06	3.87	−492.08

Models included represent the 90% confidence set (∑weight >0.90) used to calculate the model averages. k = number of model parameters, *W*_i = _Akaikés weight, Δ_i_ = evidence ratio, log (l) = log-likelihood value.

Model average-based estimates indicated that IR (β = 0.39) and host sex (β = −0.37) had effects of similar magnitude (Table 4). Although the 95% CI estimates for model averaged estimates of IR overlapped 0, they were skewed towards positive values (-0.05 to 0.84), indicating that individuals with higher IR (i.e. more homozygous individuals), harbor more species of endoparasites (Figure 2d). IR^2^ also showed a positive relationship with the endoparasite richness, but model averaged estimates indicated the magnitude of the effect on endoparasite richness was less than that of IR. The factors that better predicted endoparasite richness were: area, food, age, and year (Table 4).

### Single Locus Effects

**Figure 3:**
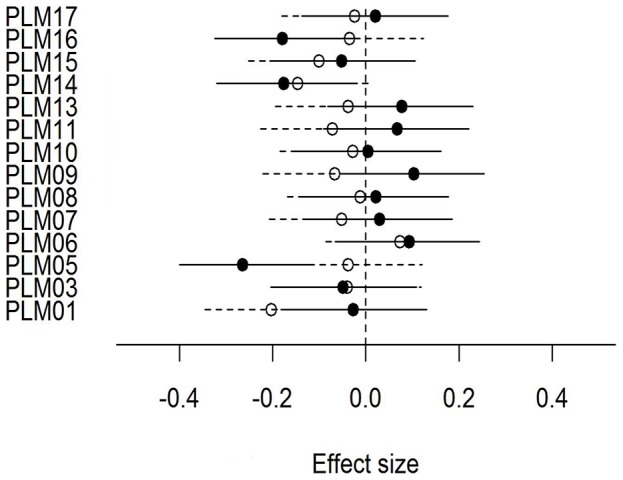
Effect sizes of single-locus for endoparasite richness and replete-ticks abundance. Effect sizes were calculated for each of the 14 microsatellite loci. Each effect size includes 95% Confidence Interval. Open circles represent the effect size for endoparasite richness and closed circles for replete tick abundance.

Three markers were significantly correlated with replete tick abundance (PLM5, PLM14, and PLM16) and 2 with endoparasite abundance (PLM01 and PLM14). However, we did not find any overall evidence for significant single locus effects, since most of the effect sizes were not significantly different from 0 (Figure 3) and the models including the single loci did not improve the variance explained by the models incorporating multi-locus heterozygosity measured as IR (F_(13,151)Endoparasites_ = 0.852, p-value = 0.604; F_(12,147)Repleteticks_ = 0.157, p-value = 0.999). However, there was a clear trend for single-locus effects to differ for ectoparasites and endoparasites. For replete ticks, effect sizes were not significantly more positive or negative (8 positive, 6 negative, p-value = 0.791), whereas for endoparasites the single locus effects were significantly more negative (1 positive, 13 negative, p-value = 0.002), suggesting that higher levels of genetic diversity are associated with reduced endoparasite richness. There was no correlation between the effect size of each marker and its genetic diversity measured either as number of alleles (r_repleteticks_ = 0.258, p-value = 0.372; r_endoparaiste_ = −0.034,p-value = 0.907), observed heterozygosity (r_repleteticks_ = 0.465, p-value = 0.093; r_endoparaiste_ = −0.142, p-value = 0.626) or expected heterozygosity (r_repleteticks_ = 0.466, p-value = 0.092; r_endoparaiste_ = −0.177, p-value = 0.544).

## Discussion

We used an information theoretic approach to assess the relative importance of genetic diversity for predicting parasitism as we simultaneously considered non-genetic factors that also influence host exposure to parasites and susceptibility. Despite the outbred character of the host study populations, genetic diversity was an important predictor of parasitism at the individual level. However, the relationship of diversity and parasitism varied in strength and shape across parasitic taxa. Endoparasite species richness displayed an inverse relationship between heterozygosity and parasitism, whereas the relationship between genetic diversity and *D. variabilis* abundance was better predicted by a curvilinear relationship, especially for replete ticks (Figure 2). In addition, we did not observe strong effects of single loci, suggesting a relationship between parasites and multi-locus diversity and supporting the idea that in some populations parasites generate selective pressure not only on the specific loci directly involved in pathogen resistance, but also throughout the genome [17], [62]. To our knowledge, this is the first example of differing relationships within the macroparasite component community in an outbred wildlife population.

**Table 4:** Model averaged estimates and odds ratios of parameters included in the 90% confidence set of models (∑weight >0.90) used to estimate endoparasite species richness.

Predictor	β(±95%CI)	SE	Odds(±95%CI)
IR	0.39(−0.05,0.84)	0.23	1.48(0.95,2.32)
IR^2^	0.21(−0.24,0.66)	0.23	1.23(0.79,1.94)
Area (Davis)	−0.08(−0.79,0.62)	0.36	0.93(0.45,1.86)
Area (PF)	−0.64(−1.58,0.30)	0.48	0.53(0.21,1.35)
Area (R)	−1.18(−2.00, −0.36)	0.42	0.31(0.13,0.70)
Area (Ref)	0.25(−0.75,1.25)	0.51	1.28(0.47,3.49)
Area (Whet)	1.13(0.29,1.98)	0.43	3.10(1.34,7.24)
Year (2007)	0.53(0.07,0.98)	0.23	1.70(1.07,2.66)
Food (Yes)	0.89(0.19,1.59)	0.36	2.43(1.21,4.90)
Age II	0.13(−0.52,0.79)	0.33	1.14(0.60, 2.20)
Age III	0.69(0.00,1.38)	0.35	1.99(1.00, 3.97)
Age IV	−0.02(−0.73,0.68)	0.36	0.98(0.48, 1.97)
Sex (Male)	−0.37(−0.81,0.08)	0.23	0.69(0.44, 1.08)

The parameters Area (Baskett), Year (2006), Food (No) and Sex (Female) were used as the reference level and model average set to 0. Davis = Davisdale CA, PF = Prairie Forks CA, R = Rudolf Bennitt CA, Ref = Reform CA, Whet = Whetstone Creek CA.

Previous studies in this population revealed that individuals that had antibodies to CDV had greater genetic diversity than individuals that were seronegative, suggesting that individuals with lower levels of genetic diversity were less likely to survive CDV [44]. Our results for endoparasite richness show a similar pattern: individuals with fewer endoparasite species had greater genetic diversity than individuals infected by more species. The most likely mechanism that might explain this pattern is that genetic variability may be important for overcoming parasitism. These HFC patterns agree with previous studies that have shown that parasites might act to maintain high genetic diversity in their host population through directional selection for heterozygous individuals [12], [14], [18], [63]. The importance of this pattern has been emphasized repeatedly in the context of inbred populations, in that directional selection imposed by parasites would select against the most inbred hosts. Due to the increase in homozygosity throughout the genome, inbred individuals are more likely to express deleterious recessive alleles, have lower probabilities of carrying adaptive alleles that may aid in infection resistance, and have a lower likelihood of heterozygosity at loci under balancing selection [18], [25]. However, our study population was not inbred. Raccoons are among the most abundant mid-sized mammals in North American temperate forests, with densities of 9–32 individuals/km^2^ at our study sites [41] and a ubiquity throughout much of North America that presumably allows high rates of gene flow. The population examined for this study showed high rates of allelic diversity and heterozygosity and significant but low F_ST_ values across sampling areas (F_ST_ = 0.008). Our results suggest that the selective pressure of parasites might also be important for populations that are not inbred and add new evidence to support the importance of genetic diversity for coping with parasitism in outbred populations [5], [14].

In contrast to the patterns observed for endoparasites and for CDV [44], the results for ectoparasites were more nuanced. Genetic diversity was not a predictor of parasitism for lice but it was a predictor of the abundance of *D. variabilis*, particularly for ticks that had been on the host long enough to obtain a blood meal. The cross-taxa differences are likely due to the different interactions of lice and ticks with the host immune system. *Trichodectes octomaculatus* is a raccoon-specific louse [64] that feeds primarily on skin debris and dried blood. As such, it is not clear whether it elicits an immune response. The suborder Ischnocera, to which it belongs, has not been shown to be affected by the host immune system in birds [47], [65]–[66] and for mammals may be better viewed as a commensal rather than a parasitic taxon [67].

Ticks, in contrast, are known to generate an immune response from the host, most prominently due to tick saliva [46], [68]. Inhibition of the host immune system has been suggested to be beneficial not only for the attachment of the tick but also for the transmission of bacteria such as *Borrellia* and *Erlichia spp*. [69]. Given the important role that genetic variability plays in facilitating the function of immune genes [15], the link between tick attachment to a host and immune system response might explain why genetic diversity is a better predictor of tick abundance than louse abundance. Furthermore, this could also explain why the strength of the relationship is greater for replete ticks, which have been exposed to a host immune response for a longer period than have non-replete ticks. We propose that the ability of a replete tick to stay attached to the host would depend on both the ability of the host to mount a successful immune response as well as the ability of the tick to modulate that immune response. As a consequence, genetic diversity is more relevant for predicting the abundance of ticks that have entered the rapid feeding stage. Interestingly, the effect of genetic diversity on ectoparasite abundance was better predicted by a quadratic measure, indicating a curvilinear relationship. Reports of quadratic effects of genetic diversity are infrequent, but have been observed for survivorship [70], reproductive success and fluctuating asymmetry [59], and most notably for louse abundance [38]. The explanation for such quadratic effects is that, given a continuum between maximal inbreeding and maximal outbreeding, there should be an intermediate level of heterozygosity that maximizes fitness [71]. However, if this applies to our results we would expect more ectoparasites on highly heterozygous or highly homozygous individuals and fewer ectoparasites on the individuals with intermediate levels of heterozygosity. This was not the case; our results show a negative relationship with the quadratic term, meaning that on average individuals that were either highly heterozygous or highly homozygous harbored fewer ectoparasites than individuals with intermediate levels of heterozygosity. A similar relationship was observed in dace (*Leuciscus leuciscus*) infected by the ectoparasite *Tracheliastes polycolpus* [72]. In that case, the authors interpreted their results as evidence for parasite-mediated disruptive selection, with both homozygous and heterozygous individuals having higher fitness than the moderately heterozygous individuals and having higher probabilities of survival.

When the relative importance of genetic diversity was compared with that of the non-genetic factors we observed different patterns across taxa that result from the specific interactions of the parasite, the environment, and the host. In previous studies we have observed that the relative importance of abiotic factors (season, year) was greater for non-replete ticks [39], the relative importance of biotic factors (age, sex and body condition) was greater for lice and replete-ticks [39], [40], and the experimental increase in social aggregation could overwhelm these factors for both taxa [41]. For endoparasites, age, sex, aggregation, and year are all important predictors of species richness [42]. With the addition of genetic diversity, however, we gain additional insights into predictors of parasitism. Relative to other factors, host genetic diversity is not important in predicting louse or non-replete tick abundance. For replete ticks, the effects of genetic diversity were comparable to those of aggregation, and more important than food. Thus, under the high contact rates induced by aggregation, the number of replete ticks that persist on a host long enough to mate and gain a full blood meal will more strongly depend on genetic host susceptibility than on age or sex, despite the fact that age and sex are themselves strong predictors of replete tick abundance in the absence of aggregation. Similarly, for endoparasite richness genetic diversity was as important as host sex. These results are especially notable given the importance that host sex and host age are often considered to have in underpinning variance in the extent of parasitism by macroparasites [1].

After analyzing the role that genetic diversity played in predicting parasitism in each group, we determined whether the effects of genetic diversity were best explained as single-locus effects or genome-wide neutral genetic diversity for endoparasite richness and replete tick abundance. We expected to find single-locus effects, since the detection of HFCs with neutral markers in non-inbred populations has usually been attributed to a particular locus [30]. In fact, it has been suggested the high polymorphism at microsatellite loci might favor the chances of one allele showing linkage disequilibrium with candidate genes [31]. However, in our study no single marker was disproportionately important in explaining any of the observed parasite-HFC models. These results agree with a recent study which suggests that frequently the effect of linked genes on HFCs are negligible compared to the effect of the rest of the genome [24]. In fact, it has been shown that the genetic architecture of resistance may be highly complex, with not only an effect of the few loci directly involved in resistance, but also strong epistatic effects between loci that can be on different chromosomes [62]. In addition, disease susceptibility might also be indirectly associated with genes involved in signaling pathways, or even metabolism [31]. Thus, it is possible that an association exists between genotypes and parasite resistance not only at the regions of the genome directly involved in resistance but in other genomic regions [17], and that here we captured this effect by using a small number of highly polymorphic microsatellites.

Interestingly, when the local effects were analyzed, they confirm the pattern found for the global HFC: for the endoparasite richness most of the local effects were negative (more heterozygous individuals presented less parasites), but for the abundance of replete ticks there is a combination of negative and positive local effects. The combination of negative and positive local effects is probably what yields a curvilinear relationship for the global HFC in replete ticks. To our knowledge, this is the first reported case where the differences are not only based on the presence-absence of an HFC with parasitism, but also on varying types of HFC relationships. The mechanism underlying these differences is unclear. We hypothesize that selective pressure would be different in different genes, with some loci being subject to overdominance that favours the heterozygotes, and other loci showing underdominance that imposes selection against heterozygotes [33], [48]. Both types of selection have been shown to act on immune genes [16], [73], and have been proposed as potential selection mechanisms underlying HFCs for single loci [33]. Whether both selection mechanisms are simultaneously acting or there are other mechanisms underlying the observed results requires further study.

The extent of parasitism of an individual is a trade-off between exposure and susceptibility. Here we have shown that even small differences in genetic diversity, such as those found in an outbred population, are associated with differences in susceptibility to parasites across individuals. Furthermore, the relationship between genetic diversity and parasitism varies across taxa, probably as a result of different selective pressures. To understand the genetic basis of parasite susceptibility, future studies must simultaneously evaluate the effects of non-genetic and genetic measures in the same models.

## Supporting Information

Table S1 **Prevalence and mean abundance for 18 species of parasites identified from raccoons in Missouri.**
(DOCX)

Table S2 **Genetic diversity measures and information for the 15 loci analyzed in an outbred raccoon population in Central Missouri (USA).**
(DOCX)

Table S3 **Ranking of models including only non-genetic terms to estimate parasite loads in a raccoon population.** (a) Abundance of replete ticks; (b) Abundance of non-replete ticks (*Dermacentor variabilis*; n = 259); (c) Abundance of lice (*Trichodectes octomaculatus*; n = 307) and (d) endoparasite richness (n = 250).
(DOCX)

Table S4 **Model averaged estimates of parameters included in the 90% confidence set of models used to estimate abundance of lice (** ***Trichodectes octomaculatus*** **).**
(DOCX)
