# Supplementary material for: The Effect and Relative Importance of Neutral Genetic Diversity for Predicting Parasitism Varies across Parasite Taxa
Source: PLoS One. 2012 Sep 26;7(9):e45404. doi: 10.1371/journal.pone.0045404 (PMC3458861; doi:10.1371/journal.pone.0045404)
Supplement: Table S1 — Prevalence and mean abundance for 18 species of parasites identified from raccoons in Missouri. (DOCX) [file pone.0045404.s001.docx]

**Table S1.**

**Prevalence and mean abundance for 18 species of parasites identified from raccoons in Missouri.**

| **Species** | **Prevalence** | **Abundance (range)** | **N** |
| --- | --- | --- | --- |
| **Ectoparasites** |  |  |  |
| *Dermacentor variabilis* | 0.96 | 23.43(0-142) | 259 |
| non-replete | 0.95 | 21.43(0-131) |  |
| replete | 0.55 | 1.99(0-24) |  |
| *Trichodectes octomaculatus* | 0.52 | 3.03(0-55) | 307 |
| **Endoparasites** |  |  | 250 |
| *Eimeria nuttalli* | 0.89 | na |  |
| *Eimeria procyonis* | 0.42 | na |  |
| *Placoconus lotoris* | 0.37 | na |  |
| *Capillaria procyonis* | 0.87 | na |  |
| *Molineus* spp. | 0.33 | na |  |
| *Baylisascaris procyonis* | 0.14 | na |  |
| *Eurytrema procyonis* | 0.29 | na |  |
| *Crenosoma* spp. | 0.17 | na |  |
| *Capillaria plica* | 0.27 | na |  |
| *Capillaria putorii* | 0.44 | na |  |
| *Physaloptera* spp. | 0.14 | na |  |
| *Atriotaenia procyonis* | 0.10 | na |  |
| *Macracanthorhynchus ingens* | 0.03 | na |  |
| *Cruzia* spp. | 0.01 | na |  |
| *Sarcocystis* spp. | 0.01 | na |  |
| *Alaria* spp. | 0.01 | na |  |

Mean abundance is presented only for ectoparasites. Ticks are subdivided into replete and non-replete individuals. Sample size (N) is the number of individual raccoons analyzed.
