# Supplementary material for: The Effect and Relative Importance of Neutral Genetic Diversity for Predicting Parasitism Varies across Parasite Taxa
Source: PLoS One. 2012 Sep 26;7(9):e45404. doi: 10.1371/journal.pone.0045404 (PMC3458861; doi:10.1371/journal.pone.0045404)
Supplement: Table S2 — Genetic diversity measures and information for the 15 loci analyzed in an outbred raccoon population in Central Missouri (USA). (DOCX) [file pone.0045404.s002.docx]

**Table S2. Genetic diversity measures and information for the 15 loci analyzed in an outbred raccoon population in Central Missouri (USA).**

| **Locus** | **GenBank Accesion no.** | **Allelic range** | **Na** | **Ho** | **He** |
| --- | --- | --- | --- | --- | --- |
| PLM01 | EF569605 | 81-101 | 11 | 0.720 | 0.731 |
| PLM03 | EF569606 | 114-156 | 11 | 0.731 | 0.729 |
| PLM05 | EF569608 | 93–127 | 14 | 0.693 | 0.707 |
| PLM06 | EF569609 | 91–123 | 11 | 0.746 | 0.773 |
| PLM07 | EF569610 | 137-169 | 14 | 0.791 | 0.833 |
| PLM08 | EF569611 | 193–215 | 11 | 0.807 | 0.819 |
| PLM09 | EF569612 | 98–130 | 14 | 0.898 | 0.890 |
| PLM10 | EF569613 | 111–133 | 12 | 0.878 | 0.873 |
| PLM11 | EF569614 | 177–187 | 6 | 0.716 | 0.691 |
| PLM12 | EF569615 | 204–222 | 10 | 0.723 | 0.790 |
| PLM13 | EF569616 | 90–132 | 19 | 0.875 | 0.905 |
| PLM14 | EF569617 | 144–164 | 11 | 0.864 | 0.883 |
| PLM15 | EF569618 | 184–202 | 10 | 0.856 | 0.827 |
| PLM16 | EF569619 | 232–242 | 6 | 0.566 | 0.587 |
| PLM17 | EF569620 | 84–102 | 10 | 0.881 | 0.862 |
|  |  |  |  |  |  |
| Mean (all loci) | |  | 11.3 | 0.783 | 0.793 |
| SD |  |  | 3.2 | 0.094 | 0.090 |
|  |  |  |  |  |  |
| Mean (without PLM12) | | | 11.4 | 0.787 | 0.794 |
| SD |  |  | 3.3 | 0.096 | 0.093 |

SD=standard deviation.
