# Supplementary material for: The Effect and Relative Importance of Neutral Genetic Diversity for Predicting Parasitism Varies across Parasite Taxa
Source: PLoS One. 2012 Sep 26;7(9):e45404. doi: 10.1371/journal.pone.0045404 (PMC3458861; doi:10.1371/journal.pone.0045404)
Supplement: Table S3 — Ranking of models including only non-genetic terms to estimate parasite loads in a raccoon population. (a) Abundance of replete ticks; (b) Abundance of non-replete ticks (Dermacentor variabilis; n = 259); (c) Abundance of lice (Trichodectes octomaculatus; n = 307) and (d) endoparasite richness (n = 250). (DOCX) [file pone.0045404.s003.docx]

**Table S3.** **Ranking of models including only non-genetic terms to estimate parasite loads in a raccoon population.**

| **Model** | **k** | **ΔAICc** | **w*_i_*** | **log (l)** |
| --- | --- | --- | --- | --- |
| (a) ***Replete ticks*** |  |  |  |  |
| Aggregation+Food+Month | 8 | 0.00 | 0.63 | -470.74 |
| Month+Age+Sex+Aggregation+Food+Body Condition | 13 | 3.05 | 0.14 | -466.81 |
| Month | 6 | 3.32 | 0.12 | -474.53 |
| Aggregation+Food | 4 | 5.60 | 0.04 | -477.76 |
| (b) ***Non-replete ticks*** |  |  |  |  |
| Month+Year+Aggregation+Food+Age+Sex | 13 | 0.00 | 0.50 | -1016.49 |
| Month+Year+Aggregation+Food | 9 | 0.41 | 0.41 | -1021.08 |
| (c) ***Lice*** |  |  |  |  |
| Aggregation+Age+Sex+Age*Sex | 10 | 0.00 | 0.43 | -579.76 |
| Aggregation+Food+Age+Sex+Body Condition+Age*Sex | 12 | 1.86 | 0.17 | -578.53 |
| Aggregation+Sex | 4 | 1.95 | 0.16 | -587.04 |
| Aggregation+Food+Age+Sex+Age*Sex | 11 | 2.15 | 0.15 | -579.75 |
| (d) ***Endoparasites*** |  |  |  |  |
| Age+Year | 6 | 0.00 | 0.35 | -502.55 |
| Age+Food+Year+Sex | 8 | 1.08 | 0.21 | -500.96 |
| Age+Food+Year | 7 | 1.08 | 0.21 | -502.03 |
| Age+Sex+Year+Aggregation+Food | 9 | 2.03 | 0.13 | -500.37 |
| Age+Food+Sex | 7 | 4.70 | 0.03 | -503.84 |

(a) Abundance of replete ticks; (b) Abundance of non-replete ticks (*Dermacentor variabilis*; n=259); (c) Abundance of lice (*Trichodectes octomaculatus*; n=307) and (d) endoparasite richness (n=250). Analyses were conducted separately for each taxon and category within taxa. Models included represent the 90% confidence set (∑weight > 0.90). k= number of model parameters, *W*_i=_ Akaike´s weight and log (l)= log-likelihood value.
