# Supplementary material for: The Effect and Relative Importance of Neutral Genetic Diversity for Predicting Parasitism Varies across Parasite Taxa
Source: PLoS One. 2012 Sep 26;7(9):e45404. doi: 10.1371/journal.pone.0045404 (PMC3458861; doi:10.1371/journal.pone.0045404)
Supplement: Table S4 — Model averaged estimates of parameters included in the 90% confidence set of models used to estimate abundance of lice ( Trichodectes octomaculatus ). (DOCX) [file pone.0045404.s004.docx]

**Table S4. Model averaged estimates of parameters included in the 90% confidence set of models used to estimate abundance of lice (*Trichodectes octomaculatus*).**

| Predictor | β (SE) | lower 95% CI | upper 95% CI |
| --- | --- | --- | --- |
| Food (Yes) | -0.12(0.26) | -0.63 | 0.39 |
| Aggregation (Yes) | -0.68(0.20) | -1.07 | -0.28 |
| Age II | 0.16(0.38) | -0.59 | 0.91 |
| Age III | -1.83(0.61) | -3.02 | -0.65 |
| Age IV | -0.57(0.41) | -1.38 | 0.24 |
| Sex (Male) | 1.75(0.34) | 1.08 | 2.42 |
| Body Condition | 0.37(0.23) | -0.07 | 0.81 |
| Age II : Sex (Male) | -0.27(0.49) | -1.22 | 0.68 |
| Age III : Sex (Male) | 1.64(0.70) | 0.26 | 3.02 |
| Age IV : Sex (Male) | 0.33(0.57) | -0.79 | 1.45 |

The parameters Area (Baskett), Year (2006), Food (No) and Sex (Female) were used as the reference level and model average set to 0.
